# Effects of prone position on the oxygenation of patients with acute respiratory distress syndrome

**DOI:** 10.1590/S1516-31802006000100004

**Published:** 2006-01-05

**Authors:** Heloisa Baccaro Rossetti, Flávia Ribeiro Machado, Jorge Luiz Valiatti, José Luiz Gomes do Amaral

**Keywords:** Prone position, Acute respiratory distress syndrome, Anoxemia, Respiratory insufficiency, Artificial respiration, Decúbito dorsal, Síndrome do desconforto respiratório agudo, Hipoxemia, Insuficiência respiratória, Respiração artificial

## Abstract

**CONTEXT AND OBJECTIVE:**

Acute respiratory distress syndrome (ARDS) is characterized by arterial hypoxemia, and prone position (PP) is one possible management strategy. The objective here was to evaluate the effects of PP on oxygenation.

**DESIGN AND SETTING:**

Non-randomized, open, prospective, controlled clinical trial, in a surgical intensive care unit at a tertiary university hospital.

**METHODS:**

Forty-one ARDS patients underwent PP for three-hour periods. Arterial partial oxygen pressure (PaO_2)_ was measured immediately before changing to PP, after 30, 60, 120 and 180 minutes in PP and 60 minutes after returning to dorsal recumbent position (DP). The paired-t and Dunnett tests were used.

**RESULTS:**

A notable clinical improvement in oxygenation (> 15%) was detected in 78.0% of patients. This persisted for 60 minutes after returning to DP in 56% and lasted for 12 and 48 hours in 53.6% and 46.3%, respectively. Maximum improvement was seen after 30 minutes in 12.5% of responding patients and after 180 minutes in 40.6%. No statistically significant associations between PP response and age, gender, weight, PEEP level, tidal volume, respiratory rate, PaO_2_/ FiO_2_ or duration of mechanical ventilation were detected. One accidental extubation and four cases of deterioration through oxygenation were detected. The 48-hour mortality rate was 17%.

**CONCLUSIONS:**

For a significant number of ARDS patients, PP may rapidly enhance arterial oxygenation and its inclusion for management of severe ARDS is justified. However, it is not a cost-free maneuver and caution is needed in deciding on using PP.

## RESUMO

**CONTEXTO E OBJETIVO:**

A síndrome de desconforto respiratório agudo (SDRA) é caracterizada por hipoxe- mia arterial e a posição em pronação (PP) é uma das estratégias de tratamento. O objetivo do trabalho é avaliar os efeitos da PP na oxigenação.

**TIPO DE ESTUDO E LOCAL:**

Estudo clínico não randomizado, aberto, prospectivo, controlado, realizado em uma unidade de terapia intensiva cirúrgica de hospital universitário terciário.

**MÉTODOS:**

41 pacientes com SDRA com idade variando entre 17 e 83 anos foram submetidos a PP durante três horas. Determinou-se a pressão parcial de oxigênio arterial imediatamente antes da mudança para PP, após 30, 60, 120 e 180 minutos em PP e 60 minutos depois de retornar para a posição dorsal (PD). Os resultados foram analisados através dos testes T pareado e Dunnett, e considerados significantes se p ≤ 0.05.

**RESULTADOS:**

Melhora clínica significativa na oxigenação (> 15%) foi detectada em 78% dos pacientes. Após 60 minutos do retorno para a posição dorsal, o efeito persistiu em 56% dos pacientes e, após 12 e 48 horas, em 53.6% e 46.3%, respectivamente. A melhora máxima ocorreu após 30 minutos apenas em 12.5% dos respondedores e em 40,6% após 180 minutos. Não foi demonstrada associação significativa entre a resposta à PP e idade, gênero, peso, nível de pressão expiratória final positiva, volume corrente, freqüência respiratória, relação PaO2/FiO2 ou duração da ventilação mecânica. Somente uma desintubação acidental e sete casos de deterioração da oxigenação foram detectados. A mortalidade em 48 horas foi de 17%.

**CONCLUSÕES:**

Em um número significativo de pacientes com SDRA, a PP pode rapidamente melhorar a oxigenação arterial e sua inclusão no tratamento da SDRA grave está justificada. Entretanto, não se trata de uma manobra inócua e cautela é necessária quando da decisão de colocar um paciente em pronação.

## INTRODUCTION

The acute respiratory distress syndrome (ARDS) is characterized by non-cardiogenic pulmonary edema, associated with changes in the ventilation-perfusion ratio, intrapulmonary shunt and arterial hypoxemia.

In such circumstances, therapeutic actions are directed towards alveolar recruitment, correction of ventilation-perfusion mismatch and shunt. Physiotherapeutic maneuvers, such as mobilization, secretion drainage and sustained pulmonary insufflation aim at reopening airways in order to facilitate pulmonary ventilation. Together with such procedures, increasing the inhaled oxygen fraction tends to diminish the shunt effect and the hypoxemia. The use of positive end expiratory pressure (PEEP) and reversal of the inspiration/expiration ratio have been associated with alveolar recruitment and arterial oxygenation improvement.

However, it must be taken into account that many of these therapeutic alternatives, along with their potential benefits, entail significant and restricting adverse effects. Utilization of high levels of PEEP may be followed by hyperinsufflation of the normal lung spaces, as a result of the intrathoracic pressure, thus hindering venous return and cardiac output. This latter effect is particularly significant when an inversion of the inspiration/expiration ratio is adopted. Decreasing the time interval intended for exhalation eventually hinders complete expiration and favors dynamic hyperinsufflation. On the other hand, increasing the inhaled oxygen fraction exposes the pulmonary parenchyma to toxic concentrations of this gas.

Since 1976, a number of researchers have proven in experimental^1-5^ and clinical^6-30^ settings that the prone position (PP) produces favorable effects regarding ventilation.

Notwithstanding advances in ARDS physiopathology and clinical investigations of the effects of positioning on pulmonary gas exchanges, there is a lack of clinical experience and information regarding this matter. Such clinical investigations would enable prediction of which patients respond to this maneuver, which ones would obtain long-lasting benefits and what the ideal length of time to keep the patient in the ventral recumbent position would be.

The current study had the aim of evaluating the effect of three hours of ventilation in PP, on the arterial oxygenation of patients affected by ARDS, and the safety of this maneuver.

## METHODS

All patients admitted to the Intensive Care Unit with the diagnosis of ARDS during six months, from October 1997 to March 1998, were prospectively screened for the protocol. ARDS was defined as a partial pressure of oxygen (PaO_2_)/fraction of inspired oxygen (FiO_2_) ratio of less than 200, with the presence of bilateral pulmonary infiltrate seen on x-ray, and in the absence of cardiogenic pulmonary edema. Mechanical ventilation was optimized after adequate sedation and neuromuscular paralysis. PEEP was adjusted in accordance with the best static lung compliance. Patients were included in the protocol if they still needed FiO_2_ of more than 0.5 in order to maintain PaO_2_ at over 80 mmHg, after the optimization of mechanical ventilation (sedation, adjustment of PEEP according to lung compliance, mobilization and secretion drainage, and manual hyperinsufflation).

Patients with unstable fractures, intracranial hypertension, severe hemodynamic instability, laparostomy or low survival expectancy were excluded.

The protocol was approved by the Research Ethics Committee of the Institution and the patients or their legal representative gave their consent.

Patients were placed in PP with the abdomen on the bed and without cushions under the shoulders or hips. Protection for the face and care regarding the catheters, tubes or probes during the maneuver were ensured. At the end of three hours, patients were put back into the supine position (SP). To evaluate the effect of ventilation in PP on oxygenation, the PaO_2_/FiO_2_ values were obtained during SP, just prior to the position change, and also after 30, 60, 120 and 180 minutes in PP and 60 minutes after returning to SP. FiO_2_ and PEEP levels were constant during the study.

Patients were considered to be responders if there was an improvement in the PaO_2_/ FiO_2_ ratio greater than 15%. This laboratory parameter alone was used to define clinically important improvement in oxygenation. All complications associated with the change in position were recorded.

Data were expressed as mean and standard deviation. The paired Student's t test and the Dunnett test for multiple comparisons were used. Results were considered significant if p < 0.05.

## RESULTS

A total of 41 patients were included (31 male and 10 female) with a mean age of 44.87 years, ranging from 17 to 83 years. Demographic data are available in Table 1.

**Table 1:** Demographics of all patients with acute respiratory distress syndrome (ARDS) included in the study

Patient number	Gender	Age (years)	Cause of ARDS	Duration of MV (days)
1	Female	74	Peritonitis	7
2	Male	52	Pneumonia (pancreatitis)	2
3	Male	78	Pneumonia	8
4	Male	56	Peritonitis	3
5	Female	61	Pneumonia (acute myeloid leukemia)	2
6	Male	47	Pneumonia	4
7	Female	35	Puerperal endometritis	6
8	Female	70	Mesenteric arterial occlusion	7
9	Male	37	Pneumonia (alcoholic cirrhosis)	1
10	Male	41	Mesenteric arterial occlusion	3
11	Female	18	Pneumonia (systemic lupus erythematosus)	8
12	Male	40	Pulmonary embolism	4
13	Male	22	Pneumonia (hepatic abscess)	1
14	Female	31	Peritonitis	1
15	Male	37	Pancreatitis	3
16	Male	32	Sepsis (hepatic failure)	1
17	Male	48	Pneumocystosis	1
18	Male	35	Sepsis (cardiac tamponade)	4
19	Male	59	Pancreatitis	5
20	Female	36	Neuroleptic malignant syndrome	1
21	Male	60	Peritonitis	8
22	Male	63	Pneumonia	6
23	Female	17	Pneumonia	12
24	Male	32	Pneumonia	1
25	Male	44	Sepsis (diabetes mellitus)	12
26	Male	32	Pneumonia (COPD)	1
27	Male	58	Sepsis	1
28	Male	23	Sepsis (cirrhosis)	1
29	Male	75	Sepsis (diabetes mellitus)	2
30	Female	55	Hepatic failure (cirrhosis, diabetes mellitus)	4
31	Male	20	Pneumonia	6
32	Female	28	Pneumonia	4
33	Male	32	Sepsis (exogenous intoxication)	4
34	Male	83	Sepsis (colon neoplasm)	9
35	Male	62	Hepatic abscess	2
36	Male	40	Multiple trauma	2
37	Male	71	Pneumonia (COPD)	4
38	Male	32	Peritonitis	3
39	Male	35	Peritonitis	7
40	Male	33	Pancreatitis	4
41	Male	36	Leptospirosis	1
Mean	–	44.87	–	5.17

*MV = mechanical ventilation, COPD = chronic obstructive pulmonary disease.*

ARDS was considered to have pulmonary origin in 43.9% of all patients. The mean PaO_2_/FiO_2_ ratio prior to being put in the prone position was 95.82 ± 38.74, ranging from 46.2 to 181.8, with a mean PEEP level of 12.43 ± 3.78, ranging from 8 to 20 cmH_2_O. The mean duration of mechanical ventilation prior to the patient's inclusion in the protocol was 5.17 days, ranging from 0.5 to 9 days.

**Figure 1:**
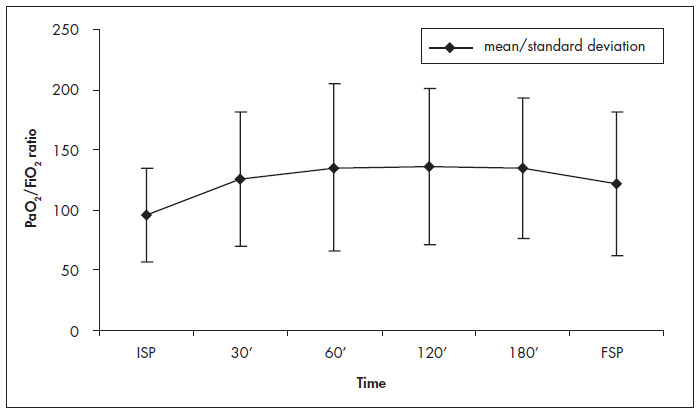
Ratio between partial arterial pressure of oxygen (PaO_2_) and fraction of inspired oxygen (FiO_2_) in the initial supine position (ISP) and final supine position (FSP) and after 30, 60, 120 and 180 minutes in prone position. Paired Student's t test results: 30 min, p < 0.0005; 60 min, p < 0.0005; 120 min, p < 0.0005; 180 min, p < 0.0005, final supine position, p = 0.003 (all values refer to comparison with initial supine position).

**Figure 2:**
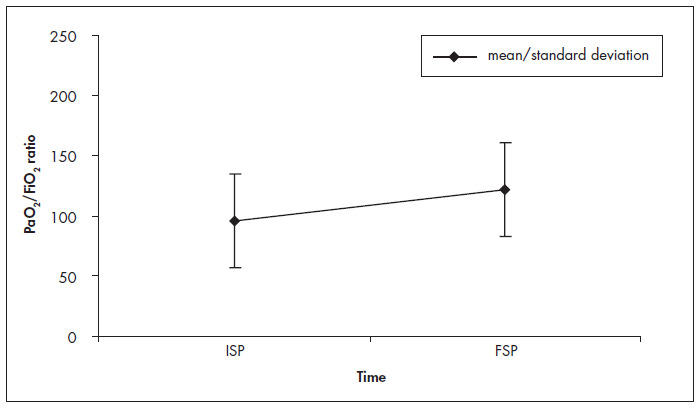
Ratio between partial arterial pressure of oxygen (PaO_2_) and fraction of inspired oxygen (FiO_2_) in the initial supine position (ISP) and final supine position (FSP) following 180 minutes in prone position. Paired Student's t test result: p = 0.003.

The mean values for the PaO_2_/FiO_2_ ratio after changing to PP were 125.79 ± 56.13, 134.90 ± 69.24, 135.60 ± 64.93, 134.50 ± 58.25 and 121.60 ± 60.00 after 30 min, 60 min, 120 min, 180 min and the return to SP, respectively (Table 2). There was a significant difference between the prior PaO_2_ /FiO_2_ ratio and PP after 30 min (p < 0.0005), 60 min (p < 0.0005), 120 min (p < 0.0005), 180 min (p < 0.0005) and SP (p = 0.003). These results were confirmed by the multiple comparison test (Figures 1 and 2).

**Table 2:** Ratio between partial arterial pressure of oxygen (PaO_2_) and fraction of inspired oxygen (FiO_2_) in the initial supine position and after 30, 60, 120 and 180 minutes in prone position, and in the final supine position

Patient	Initial supine position	Prone position				Final supine position*
		30 min	60 min	120 min	180 min	
1	68.1	74.4	69.6	**87.2†**	**78.7**	**81.3‡**
2	96.7	108.8	**136.5†**	110.4	110.1	114.6‡
3§	136.0	148.2	144.0	148.3	147.6	141.0
4	72.3	**110.5**	**124.9**	**173.6**	**174.6†**	**135.5‡**
5	50.2	**83.2**	**92.8**	**93.2†**	**58.6**	50.9
6	46.2	**57.9**	**54.8**	**57.2**	**61.1†**	52.9
7	50.0	**74.0**	**80.0**	**96.0†**	**80.0**	56.0
8	51.0	54.0	**59.0**	46.0	49.0	**59.0‡**
9^§^	77.0	63.0	55.0	54.0	47.0	82.0
10	102.0	**179.0†**	**145.0**	**174.0**	**178.0**	**178.0‡**
11^§^	88.2	46.0	53.0	55.0	55.0	71.0
12	54.0	**80.6†**	**72.1**	**68.5**	**77.0**	**76.6‡**
13	174.2	**241.2†**	**203.6**	**216.9**	**241.2**	98.0
14	128.9	**164.6**	**197.2**	**213.4†**	**164.5**	**148.4‡**
15	118.0	**139.5**	89.5	118.6	**142.8†**	131.0
16§	70.0	65.5	74.6	77.1	80.0	67.0
17	181.8	**228.1**	**241.5**	**241.4**	**249.7†**	**216.0‡**
18§	180.1	105.4	142.8	180.1	171.9	**253.9**
19§	149.0	81.7	96.0	93.0	86.0	111.0
20	98.0	**181.1**	**215.0†**	**127.0**	**135.0**	68.0
21	112.0	**214.0†**	**162.0**	**144.0**	**149.0**	109.9
22	118.0	**205.5**	**263.8†**	**248.6**	**231.9**	116.0
23	52.0	**85.3**	**81.2**	**69.0**	**88.0†**	53.0
24	114.5	**173.4**	**187.8†**	**185.6**	**168.9**	193.4‡
25	90.7	103.0	**135.4**	**151.3**	**160.3†**	**107.6‡**
26	51.0	52.0	50.0	52.0	**63.0†**	46.0
27^§^	93.9	90.0	84.9	83.0	94.5	92.0
28	103.4	**190.0**	**175.0**	**179.5**	**227.0†**	**159.0** ‡
29§	157.7	108.7	126.3	101.3	111.3	110.0
30	87.7	**134.1**	**131.1**	**139.0†**	**128.0**	**139.4‡**
31	52.7	**133.0**	**226.0**	**227.0†**	**200.0**	**231.0‡**
32	146.2	**169.4**	**190.0**	**180.2**	**214.0†**	**182.4‡**
33	57.3	**110.0**	**97.9**	**111.6**	**140.4†**	**76.0‡**
34	54.0	**87.0**	61.0	**105.1**	**179.0†**	**188.2‡**
35	88.5	**123.8**	**147.5†**	**116.3**	101.3	**106.3‡**
36	101.3	**162.5**	**173.8**	**182.5**	**185.0†**	106.3
37	120.0	**218.0**	**312.0†**	**272.0**	**256.0**	**154.0‡**
38	113.0	**229.0**	**301.0†**	**284.0**	**204.0**	**311.0‡**
39	103.0	**121.0**	108.0	**146.0†**	115.0	**139.0‡**
40^§^	72.0	69.0	68.0	56.0	68.0	**93.0**
41	48.0	**92.0**	**101.0**	**96.3**	**143.0†**	**81.0‡**
Mean ± standard deviation	95.82 ± 38.74	**125.79 ±56.13**	**134.90 ±69.24**	**135.6 ±64.93**	**134.50 ±58.25**	**121.60 ±60.00**

*In boldface: PaO_2_/FiO_2_ ratio more than 15% greater than initial value;*

* *final supine position: samples were collected 60 minutes after returning to supine position (following 180 minutes in prone position);*

† *maximum improvement in prone position;*

‡ *continuing responders;*

§ *non responders.*

*Paired Student's t test: 30 min, p < 0.0005; 60 min, p < 0.0005; 120 min, p < 0.0005; 180 min, p < 0.0005; supine final, p = 0.003 (all values in comparison with initial supine position).*

A clinically important improvement in oxygenation (improvement in PaO_2_/FiO_2_ ratio > 15%) was detected in 32 patients (78.0%) (Table 2). In 65.85%, such improvement took place within the first 30 minutes of PP. However, only 12.5% of the responders showed their maximum improvement within these first 30 minutes. For 25.0%, 21.8% and 40,6% of the responders, the maximum improvements were achieved after 60 min, 120 min and 180 min, respectively (Table 2).

Among the responders, 21 (65.6%) were considered to be continuing responders, since the PaO_2_/FiO_2_ ratio remained at least 15% higher than the previous supine levels even after returning to SP (Table 2). Moreover, 70.0% (14 patients) of these continuing responders maintained at least this level of improvement (15%) for 24 hours and 50.0% (10 patients) for 48 hours after the maneuver. Four of these patients received nitric oxide due to refractory hypoxemia.

Nine patients were considered to be nonresponders. Two patients had no clinically significant alteration in oxygenation after PP. In seven patients (17.07), the oxygenation deteriorated in PP, with a mean decrease in the PaO_2_ /FiO_2_ ratio of 34.71%, considering the worst value regardless of the length of time in PP. Four of them (9.75%) did not improve again after returning to SP (Table 2).

The maneuver was relatively easy to perform, and only one patient had an accidental extubation (2.4%). Another especially noticeable complication was facial edema with no additional morbidity. Eight deaths (19.5%) occurred within the first 48 hours following the study procedure, but were unrelated to the maneuver. These patients had severe ARDS, with a mean PaO_2_ /FiO_2_ ratio of 66.79 ± 25.23.

The duration of mechanical ventilation, age, weight, baseline PaO_2_/FiO_2_ ratio, tidal volume, respiratory rate and level of PEEP prior to changing the position did not seem to influence the response to this maneuver, although there was a trend towards higher weight among the responders: 74.6 ± 14.4 kg and 65.0 ± 8.8 kg, for the responders and non-responders, respectively (p = 0.058; Table 3).

**Table 3:** Comparison between responders and non-responders to prone positioning

Variable	Non-responders	Responders	p*
Age (years)	47.9 ± 20.6	43.8 ± 16.8	0.48
Weight (kg)	65 ± 8.8	74.6 ± 14.4	0.058
PaO_2_/FiO_2_ ratio†	102.4 ± 45.4	93.4 ± 36.6	0.52
Tidal volume	490.0 ± 119.2	536.7 ± 143.4	0.34
Respiratory rate	15.3 ± 2.8	15.2 ± 3.3	0.92
PEEP (mmHg)	12.7 ± 4.8	12.3 ± 2.9	0.71
Duration of MV (days) ‡	3.8 ± 2.9	3.8 ± 2.8	0.96

*PaO_2_/FiO_2_ ratio: ratio between partial arterial pressure of oxygen and fraction of inspired oxygen. PEEP: positive end expiratory pressure. MV: mechanical ventilation.*

* *Student's t test. All variables are expressed as mean ± standard deviation;*

† *initial supine position;*

‡ *before prone positioning.*

## DISCUSSION

The treatment of ARDS is based on oxygen therapy and ventilation under positive pressure, with high levels of positive end expiratory pressure. These procedures are restricted by the risks of oxygen toxicity and barotrauma.^31-33^ In this context, the prone position could enable the use of lower fractions of inhaled oxygen and lower airway pressures. Moreover, lung inflation is more homogeneous in PP, contributing towards reduction in the risks of ventilator-induced lung injury.

A series of researchers have detected optimized oxygenation after changing from SP to PP that may be clinically significant and persistent in the majority of such patients.^6-30^ These observations, allied to the simplicity of the procedure and its low incidence of significant adverse effects, have justified the inclusion of PP in the routine treatment for hypoxia associated with ARDS. Since 1998, we have been including the change in recumbent position from supine to prone within the strategy for severe hypoxemia treatment (PaO_2_ /FiO_2_ < 200 mmHg, with optimized PEEP). Except in certain situations that constrain this maneuver (intracranial hypertension, unstable fractures, peritoneostomy and severe hemodynamic instability), ventilation in PP has been performed frequently.

In fact, ventilation in PP does not entail major technical difficulties. Other than the few minutes needed for changing between the types of recumbent position, adoption of the PP procedure does not substantially change the routine of medical or nursing care. Sedation is required, but it is also a requisite for the ventilation of severely hypoxemic patients.

Alternation of the recumbent position requires special attention to the placement of tubes, probes and catheters, the protection of areas at risk of injuries due to compression (eyes and other structures of the face, upper limbs and genitals), the attachment of the monitoring systems and connections of the ventilation circuit. Although these 41 patients were lying down on the bed, some authors have suggested that the abdomen should be kept free from the bed surface, by putting cushions under the shoulders and anterior iliac crests. This would facilitate expansion of the thoracic cage and diaphragm excursions.^3^ However, other authors have discarded such an approach, and have simply put their patients lying down on the bed.^4,6,12^

In our study, patients were considered to be responders if there was an improvement in the PaO_2_ /FiO_2_ ratio greater than 15%. This was also considered to be a clinically significant improvement. This value was chosen on the basis of the literature, in which other authors have used changes of 10 to 20% in this ratio, or an absolute improvement of 20 mmHg in PaO_2_, to define which patients were responders to PP.^6,7,17,23,27^ Using this figure of 15%, most of our patients (78.0%) did improve their oxygenation in PP as early as 30 minutes after the maneuver (65.85% of all patients). Moreover, 62.5% had a continuing response even after changing back to SP. This has already been shown by other studies.^6-30^ Moreover, this has been demonstrated in different settings, such as pulmonary aspi- ration,^15^ non-ARDS patients,^16^ hydrostatic pulmonary edema^17^ and both in pulmonary and extra-pulmonary ARDS.^18^ Many recent reviews have also pointed out that this maneuver is associated with an improvement in oxygenation.^34-36^

However, one recent multicenter trial,^37^ with more than 300 patients randomized to be in PP for 6 hours or to remain in SP, failed to show reduced mortality in the six-hour PP group. Although it has not yet been proven that the prone position has an impact on ARDS survival, this maneuver is clearly associated with an improvement in oxygenation, as prone-positioned patients had a significant improvement in the PaO_2_/FiO_2_ ratio and in the tidal volume. It is possible that the disappointing results regarding mortality in that study were due to the short time spent in the prone position. In that paper, there was no information regarding the maximum improvement in the prone position over the course of time. This means that it is unknown how many patients were still improving their oxygenation at the end of the prone period. In our study, we have shown that, although 65% of the patients did improve within the first 30 minutes in PP, there was a continuous improvement in oxygenation, with the majority of the patients achieving the maximum PaO_2_ /FiO_2_ ratio in the third hour after prone. This means that they were still improving at the end of the three hours, thus suggesting that a longer period of PP would be needed to achieve the maximum response. This was also suggested by a recent study in which 11 patients put in the prone position for up to 18 hours did show a continuous improvement in oxygenation.^19^ Unfortunately, this latter study was not a controlled trial, so it is possible that the patients improved for reasons other than PP. Nonetheless, it is possible that keeping the patients in PP for a longer time would lead to greater benefit in terms of mortality.improvement in the PaO_2_/FiO_2_ ratio and in the tidal volume. It is possible that the disappointing results regarding mortality in that study were due to the short time spent in the prone position. In that paper, there was no information regarding the maximum improvement in the prone position over the course of time. This means that it is unknown how many patients were still improving their oxygenation at the end of the prone period. In our study, we have shown that, although 65% of the patients did improve within the first 30 minutes in PP, there was a continuous improvement in oxygenation, with the majority of the patients achieving the maximum PaO_2_ /FiO_2_ ratio in the third hour after prone. This means that they were still improving at the end of the three hours, thus suggesting that a longer period of PP would be needed to achieve the maximum response. This was also suggested by a recent study in which 11 patients put in the prone position for up to 18 hours did show a continuous improvement in oxygenation.^19^ Unfortunately, this latter study was not a controlled trial, so it is possible that the patients improved for reasons other than PP. Nonetheless, it is possible that keeping the patients in PP for a longer time would lead to greater benefit in terms of mortality.

Another possible explanation for the absence of impact on survival lies in the fact that it might be difficult to show such an impact since hypoxemia is not a major cause of death among ARDS patients. Most of these patients die from multiple organ dysfunction syndrome and the main benefit of this maneuver is to allow better oxygenation. In another trial,^37^ patients were put in the prone position except if the PaO_2_ /FiO_2_ ratio was greater than 200 with a PEEP level of 5. This meant that, even if less critically ill patients were included, which may have contributed to the absence of any effect on survival, the effect on oxygenation could still be demonstrated. This had already been shown in another study in which patients with acute lung injury (ALI) showed an improvement in oxygenation.^16^ If only very critically ill patients had been included in the Gatinonni trial,^37^ the results relating to mortality might have been different, since this maneuver undoubtedly increases oxygenation, and this may have been more important among critically hypoxemic patients. This was suggested because, in the subgroup of patients with the lowest PaO_2_/FiO_2_ ratio, there was a significant reduction in mortality.

This concept is important, as it is not a cost-free maneuver. Although in the literature the prone position has not been associated with major adverse events, we found a small percentage of patients that had a clinically important and persistent deterioration of saturation. In three instances (cases 9, 18 and 40), returning the patients to the dorsal recumbent position reversed this hazard, thus suggesting that PP was responsible for the deterioration in oxygenation. Worsening of oxygenation after putting the patient into PP is not a frequent finding in the literature. In one study, a patient died soon after being placed in SP because of oxygenation deterioration in PP, and this was considered to be an adverse event related to the maneuver.^16^ It is possible that this situation has been underestimated in other trials and therefore its importance is now underscored.

Another concern is the episode of accidental extubation in our sample. This is a troublesome situation, since our intensive care unit (ICU) team is well trained and very used to the maneuver. Even in this setting, accidents may happen and, because of the severity of these patients, extubation might be responsible for an irreversible deterioration. Our patients, although relatively young, were critically ill, as shown by the low PaO_2_ /FiO_2_ ratio, high PEEP levels and the high acute mortality rate (19% within 48 hours). Since most of them presented hypoxemia or needed high FiO_2_ levels, it seemed reasonable to put them in PP, regardless of the potential danger that it could cause. As there is no proven benefit in terms of mortality, patients that are not critically ill should not be routinely put in PP.

Unfortunately, we could not find a recognizable characteristic or marker to determine whether or not a patient would be a responder or, even more importantly, would deteriorate in PP. Until we can identify which patients should be put into the prone position, it is important to promptly recognize this subgroup of nonresponding patients, so that the maneuver can be interrupted immediately. This is reinforced by the fact that this maneuver, at least from the evidence available, has no impact on mortality. The cost-benefit ratio in this subgroup of patients is fairly discouraging.

## CONCLUSION

The analysis of the results achieved in this study leads us to conclude that, in a considerable number of patients with ARDS, the change from the SP to the PP is responsible for a sustained improvement in arterial oxygenation. In some patients, however, worsening follows the change in position and so far it has not been possible to identify such patients. Since there is no proven impact on mortality, ventilation in PP is potentially beneficial and deserves to be taken into account for the treatment of severe hypoxemia associated with ARDS. For other cases, it needs to be used with caution.
